# Case report: A novel *APTX* p.Ser168GlufsTer19 mutation in a Chinese family with ataxia with oculomotor apraxia type 1

**DOI:** 10.3389/fneur.2022.873826

**Published:** 2022-09-01

**Authors:** Xuan Wu, Nan Dong, Zhensheng Liu, Tieyu Tang, Meirong Liu

**Affiliations:** ^1^Department of Neurology, The First Affiliated Hospital of Soochow University, Suzhou, China; ^2^Department of Neurology, Affiliated Hospital of Yangzhou University, Yangzhou, China

**Keywords:** ataxia with oculomotor apraxia type 1, oculomotor apraxia, APTX, novel mutation, case report

## Abstract

Ataxia with oculomotor apraxia type 1 (AOA1) is a rare genetic disorder and is inherited in an autosomal recessive manner. It is mainly characterized by childhood-onset progressive cerebellar ataxia, with dysarthria and gait disturbance being the two most common and typical manifestations. Axonal sensorimotor peripheral neuropathy, dystonia, chorea, and cognitive impairment are common associated symptoms, as are hypoalbuminemia and hypercholesterolemia. Oculomotor apraxia (OMA)has been reported to be a feature often, although not exclusively, associated with AOA1. The Aprataxin gene, *APTX*, is ubiquitously expressed, and numerous APTX mutations are associated with different clinical phenotypes have been found. In the present study, we enrolled a 14-year-old boy who developed ataxia with staggering gait from the age of 4 years. Early-onset cerebellar ataxia, peripheral axonal neuropathy, cognitive impairment and hypoalbuminemia, hypercholesterolemia were presented in this patient, except for OMA. We applied ataxia-related genes filtering strategies and whole-exome sequencing (WES) to discover the genetic factors in a Chinese family. Sanger sequencing was used in the co segregation analysis in the family members. A compound heterozygous mutation in APTX gene (c.739C>T and c.501dupG) was identified. This is the first description of a genetically confirmed patient of AOA1 in a Chinese family in addition to a novel mutation of c.501dupG in APTX.

## Introduction

Autosomal recessive cerebellar ataxia (ARCA) is a complex, heterogeneous, and disabling neurogenetic degenerative disease characterized by progressive damage to the cerebellum and its associated conduction tracts (1). Early-onset AOA1 is a slowly progressive cerebellar ataxia, which usually occurs in childhood, followed by oculomotor dysfunction and peripheral axonal neuronal lesions (2). Patients will have symptoms of disappearance of deep tendon reflexes, peripheral nervous system lesions 7–10 years after onset, limb atrophy, chorea, dystonia and other phenomena, as are hypoalbuminemia and hypercholesterolemia in the course of the disease. Some patients have cognitive impairment, although intellect remains normal (3, 4). AOA1 is the most common ARCA in Japan (5), and is also found in other countries (6, 7). OMA is a characteristic ocular symptom, manifested as abnormal smooth eye tracking saccade, disappearance or defect of voluntary eye movement in the horizontal direction, and normal eye movement in the vertical direction. Some patients have strabismus or blinks are often seen (8), which is the prominent clinical presentation in AOA1.However,the AOA1 patients with absence of OMA were reportedly presented in 34.5% cases (9), so these patients experience genuine OMA remains questionable.

In this study, we report a childhood-onset AOA1 patient who presented with progressive cerebellar ataxia, and peripheral neuropathy, but no oculomotor apraxia. The patient underwent clinical and molecular genetic testing, which WES identified a compound heterozygous mutation of c.739C>T and c.501dupG in *APTX*. This is the first report of the *APTX* c.501dupG mutation. Sanger sequencing was used in the cosegregation analysis in the family members. Our study expanded the variant spectrum of the APTX gene and contributed to genetic counseling and prenatal genetic diagnosis of the family. Currently, there is no effective treatment is available forAOA1 in general. Different forms of rehabilitation remain supportive and symptomatic, with the main objective of improving the quality of life of patients.

## Case report

We report a 14-year-old male patient who has experienced progressive gait disturbance characterized by difficulty in maintaining balance since the age of 4 years. He is the first child of healthy, non-consanguineous Chinese parents with no family history of gait disorders. The boy was referred to have had normal development until 4 years of age, when parents started noticing unsteady gait with frequent falling down, cognitive, and speech worsening. At age 7 years he presented ataxia increasing with effort, dysarthria, gait disorders, slowness, learning difficulties, exercise intolerance and fatigue needing to rest. The boy at the age of 10 years was admitted for his first neurological examination, which revealed markedly cerebellar syndrome with gait ataxia and dysarthria attention, and mild cognitive and executive function decline. Physical examination revealed pes cavus, but no scoliosis or other musculoskeletal deformities. Deep tendon reflexes of the lower extremities were disappeared and the plantar response was flexor bilaterally. Light touch, pinprick, and vibration sensation were absent, and the Romberg test was positive. Neurological examination was characterized by gait imbalance and absence of dystonia, chorea, tremor and dyskinesia (Supplementary Video 1). His extraocular range of motion was adequate in all directions, with no apparent OMA on reflex saccade examination. The boy at the age of 14 was screened by a trained neurologist and were noted a mild cognitive impairment. The clinical manifestations were consistent with AOA1.

**Figure 1:**
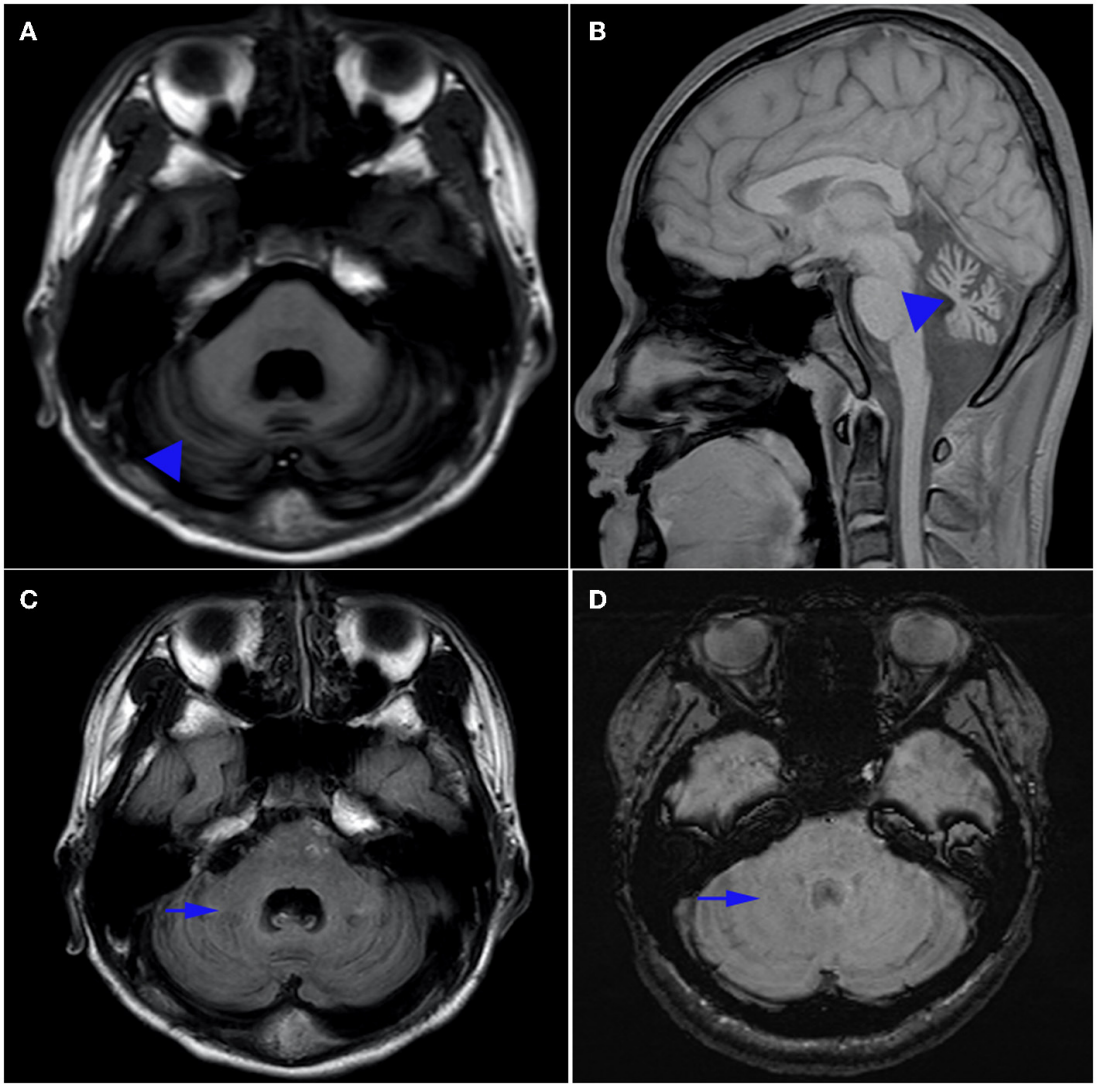
Brain MRI. Axial T1-weighted **(A)** and sagittal T1-weighted **(B)** magnetic resonance images of the patient. Note the presence of pure cerebellar atrophy and low cerebellar dentate nucleus signal, without involvement of the brain stem and cerebral cortex. Hyposignal in the dentate nucleus can be seen on T1 **(C)**, but is absent on susceptibility weighted imaging (SWI) **(D)**.

Biomedical tests for ceruloplasmin, serum immunoglobulin, alpha-fetoprotein, ferritin, urethane lactate, pyruvate, liver and kidney function, electrolytes, hemoglobin, vitamin E and B12 were performed. No results were abnormal except for hypoalbuminemia (30.5 g/L, normal range 40–55 g/L) and mildly elevated cholesterol (6.1 mmol/L, normal range <5.2 mmol/L). Serum alpha-fetoprotein (AFP) level was normal. The patient's visual evoked potentials were abnormal in low-amplitude responses on binocular and monocular tests, although it was not appreciated on history or physical examination. Nerve conduction study showed reduced nerve conduction velocities and low sensory and motor action potentials indicating sensorimotor axonal polymorphic peripheral nerve damage. The lower extremity muscles were biopsied, and histological analysis showed that the size of muscle fibers varied slightly, showing neurogenic pathological changes. Magnetic resonance imaging (MRI) of the head showed atrophy of the cerebellum with no involvement of the brainstem or cerebral cortex, and low signal loss in the cerebellar dentate nucleus on sensitivity-weighted imaging (SWI) (Figure 1). MRI scan was evaluated by an experienced radiologist. Written informed consent was obtained from the parents for publication and accompanying images.

Given the tentative diagnosis of cerebellar ataxia, genetic evaluations for spinocerebellar ataxia (SCA1, 2, 3, 6, 7, 8, 10, 12, 17, 36), dentatorubral-pallidoluysian atrophy (DRPLA), and the autosomal recessive ataxias Friedreich's ataxia (FRDA) were performed and shown to be negative.

**Figure 2:**
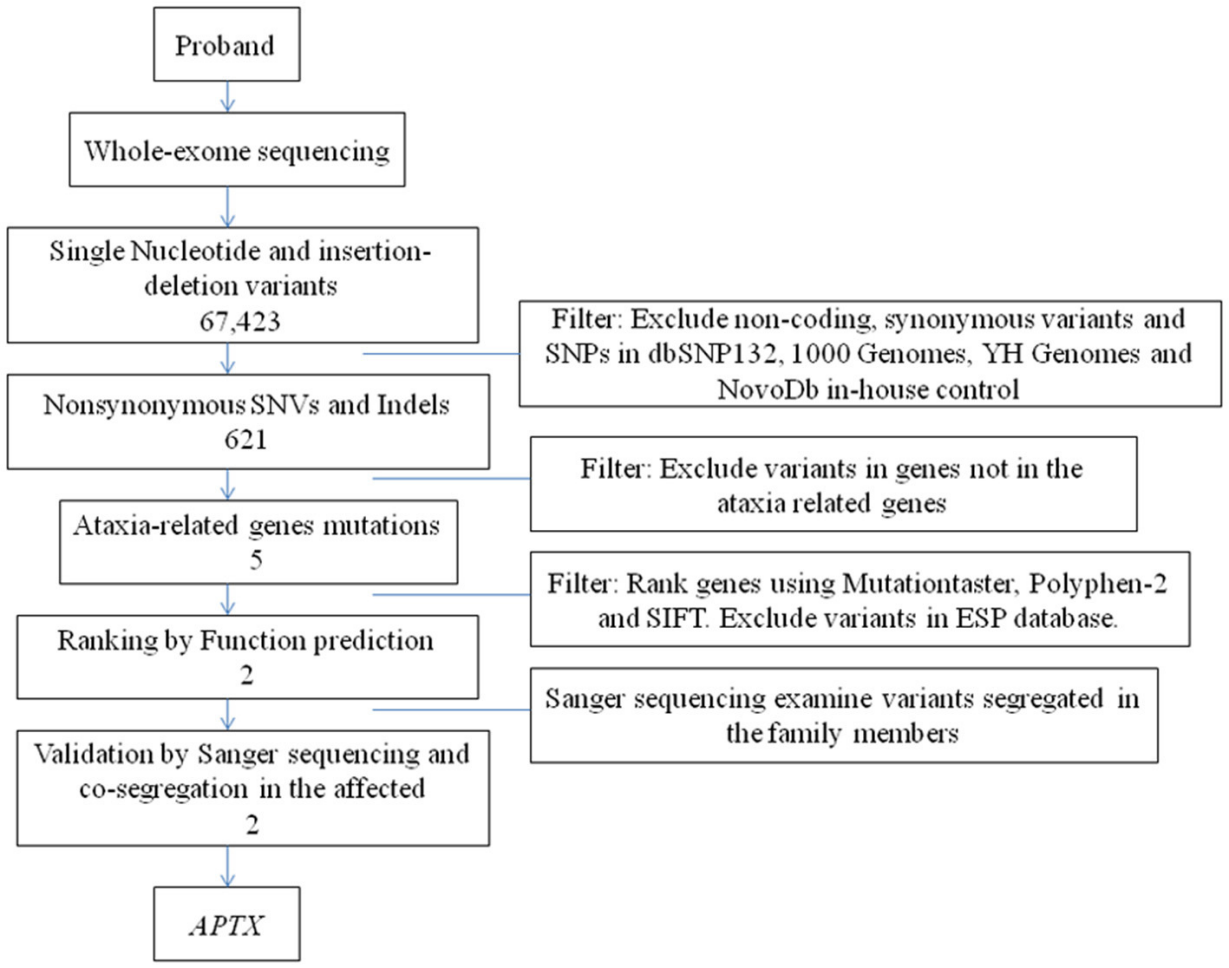
The strategies of gene date filtering.

We then performed WES to identify the genetic lesions responsible for the disease phenotype of the proband. The main part of WES was provided by the Novogene Bioinformatics Institute (Beijing, China). The exomes were captured using Agilent SureSelect Human All Exon V6 kits (Agilent Technologies, Sta Clara, CA, USA), and high-throughput sequencing was performed using Illumina HiSeq X-10 (Illumina, San Diego, CA, USA). The basic bioinformatics analysis including Reads, Mapping, Variant detection, Filtering, and Annotation were also endowed by Novogene Bioinformatics Institute. The strategies of data filtering refer to Figure 2. Sanger sequencing was applied to validate the candidate variants identified in whole-exome sequencing. Cosegregation analysis was conducted in all family members of this study. The primers were as follows: c.739C>T (forward: 5′-AAGTCAGGCAGAGAGGTGGA−3′, reverse: 5′-CGTTACCATTGGCTGGTCTT−3′); c.501dupG (forward: 5′- CAAGGCAGAGGGGATATTCA−3′,reverse: 5′- GAGGCAGGAGAATCACTTCG−3′), and the sequences of the polymerase chain reaction (PCR) products were determined using the ABI 3100 Genetic Analyzer (ABI, Foster City, CA).

**Figure 3:**
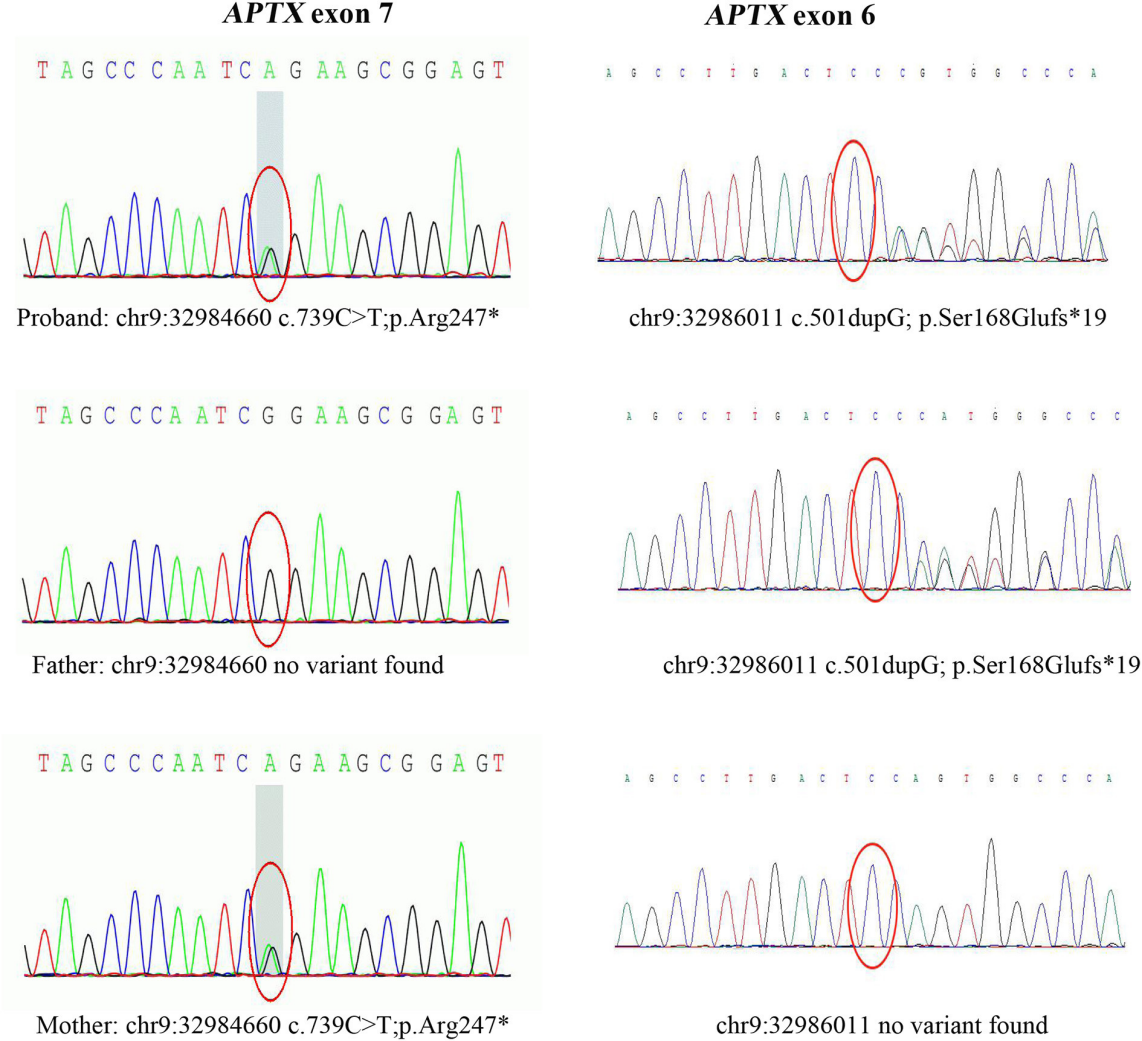
Sanger sequencing of *APTX* exons. Sanger sequencing after PCR amplification revealed compound heterozygous mutations: the nonsense mutation (c.739C>T, p.Arg247*) in exon 7, and a frameshift mutation (c.501dupG, p.Ser168Glufs*19) in exon 6. c.501dupG is novel, while c.739C>T is a known pathogenic mutation.

After data filtering, a nonsense mutation in exon 7 (c.739C >T; p.Arg247^*^) and a frameshift mutation in exon 6 (c.501dupG; p.Ser168Glufs^*^19) were identified by using WES,and they were co-segregated with the affected members (Figure 3). The patient's father carried a single heterozygous mutation (c.501dupG), and his mother carried a single heterozygous mutation (c.739C>T). Mutation in APTX c.501dupG, which has not been reported previously, located at Chr9:32,986,011(Exon 6).

Currently, the patient has accepted medicine (Idebenone20 mg daily and CoQ10 30 mg daily) and supportive procedures including rehabilitation therapy, and speech therapy. However, the boy experienced no significant clinical improvement following the procedure.

## Discussion

In this study, we applied WES to detect the underlying genetic cause in a Chinese patient with AOA1. A new frameshift mutation (c.501dupG/p.S168Efs) of APTX was identified. The APTX gene, mapped to chromosome 9p13.3, encodes Aprataxin nuclear protein, present in both the nucleoplasm and the nucleolus. Aprataxin is a member of the histidine triad (HIT) superfamily and includes three domains: a forkhead-associated domain in the N-terminal region; a HIT domain, and a C-terminal region containing a divergent zinc finger motif (10). Aprataxin interacts with a variety of proteins involved in the RNA-DNA damage response to protect the genome from adenosyl ribosylation and mitochondrial transcriptional regulation (11–13). Changes in the structure of the Aprataxin protein affect DNA repair, resulting in the gradual accumulation of unrepaired DNA strand breaks cellular dysfunction, especially in the degeneration of Purkinjie and cerebellar granular cells, which are responsible for cerebellar atrophy and ataxia in AOA1 (14, 15). At present, at least 40 mutations of APTX have been reported in patients according to Human Gene Mutation Database (HDMG), mainly including nonsense, splice site, missense, frameshift and deletion mutation (16, 17). The phenotypes resulting from these mutations are observably heterogeneous, mainly including progressive ataxia, OMA, chorea, dystonia, and peripheral neuropathy, hypoalbuminemia, hypercholesterolemia (4). The relationship between genotype and early-onset ataxia and OMA is not completely clear. Previous studies (5) proposed insertion, deletion, frameshift mutation in APTX may cause a more severe phenotype with childhood onset compared with phenotypes caused by missense mutations with relatively late age at onset (9). Missense mutations causing mild phenotypes are in or downstream of the HVHLH motif in the HIT domain, while others are in the first part of the HIT domain (18). The HIT domain shares residues 173–273 amino acid length of Aprataxin protein 7) and our exome-sequencing analysis reports a compound heterozygous mutation of c.739C>T(p.R247^*^) and c.501dupG(p.S168Efs^*^) located in the HIT domain region, which is predicted to cause truncated protein ranging from HIT to zinc finger domain. Mosseso et al. (19)confirmed that an AOA1 patient with a mutation c.739C>T of the APTX gene leading to the nonsense variation R247X and to a truncated product with deletion of the HIT and zinc-finger domains of the protein, but they failed to elaborate on the patient's clinical phenotype. We identified a new mutation c.501dupG, resulting in a frameshift with a premature stop, which is predicted to truncation of functional domains of Aprataxin protein. The position of pathogenic mutants related to the HIT domain could be one of the factors contributing to the clinical phenotype in AOA1 and should be further evaluated in future studies. Our patient had onset at age 4 years of progressive gait ataxia, dysarthria and peripheral neuropathy, but had normal ocular movements, so we highlight AOA1 should be included in the differential diagnosis of early-onset ataxia even in the absence of OMA. The small numbers of individuals with identical *APTX* mutations, the great diversity in background genotypes and the substantial phenotypic heterogeneity has limited the ability to determine the impact of clinical outcomes. Consequent more data and research are required to provide further clues.

AOA1 typically manifests with early-onset cerebellar ataxias predominate at onset, and often masked by severe axonal sensorimotor neuropathy ultimately leading to amyotrophy occurs in the majority of patients (3, 4). The age of our patient at onset was 4 years old and a slow progression that results in a spectrum of severity from progressive gait instability, to patients becoming wheelchair bound from the age of 12, which is consistent with the age spectrum of AOA1. Hypoalbuminemia and hypercholesterolemia found in 83 and 75% of patients, are the most important clues to AOA1 diagnosis (4). Normal AFP levels distinguish it from ataxia telangiectasia (AT) and AOA2 and to some extent in ARCA3 (1, 20). Movement disorders were absent in our patient. Yokoseki et al. found that chorea, dystonia, and myoclonus accounted for 40, 20, and 10% of movement disorders respectively. Different mutations in APTX gene have different molecular mechanisms as evidenced by the varying phenotypes resulting from mutations affecting different domains of the same Apatataxin protein, but the majority of these mechanisms remain to be determined.

In our study, there was atrophy of the cerebellum with no involvement of the brainstem or cerebral cortex in T1 sequences of magnetic resonance image. Magnetic resonance (SWI) showed the absence of low signal in the cerebellar dentate nucleus which is especially sensitive to iron and other metal stores and can be used to assess changes in iron content in different neurodegenerative diseases (21). The findings indicated that SWI was a sensitive technique for detection of cerebral clinic anatomical correlations in AOA1 patients (22).Anatomical experiments had provided evidence for dentate nucleus and striatum as well as the subthalamic nucleus and cerebellar cortex may be damaged (23). So the disappearance of low signal intensity in the dentate nucleus on SWI and the persistence of T1 sequences (24) suggest that the underlying mechanism of AOA is not only cerebellar atrophy, but also dentate atrophy, especially changes in iron content in nucleoids.

There is no effective treatment and current treatment strategies are based on rehabilitation therapies, including special strength and balance training to increase stability as well as compensatory strategies (25). In the next decade, novel treatments might target in pathogenesis, including mitochondrial dysfunction, impaired DNA repair, by modulation of gene expression, stem cell transplantation, gene transfer, or interventions in viral pathways (26).

## Conclusion

In summary, the present study has confirmed a compound heterozygous mutation of c.739C>T and c.501dupG in APTX in a Chinese patient, in addition to a novel mutation of c.501dupG. The patient presented with AOA1 phenotypes including childhood progressive cerebellar ataxia, cognitive impairment and peripheral neuropathy, but no oculomotor apraxia. We highlight the genetic and phenotypic heterogeneity of AOA1, such as the lack of typical OMA features. Our results expand on the spectrum of APTX mutations and contribute to the genetic diagnosis and counseling of families with AOA1.

## Data availability statement

The datasets presented in this article are not readily available because of ethical and privacy restrictions. Requests to access the datasets should be directed to the corresponding author/s.

## Ethics statement

Ethical review and approval was not required for the study on human participants in accordance with the local legislation and institutional requirements. Written informed consent to participate in this study was provided by the participants' legal guardian/next of kin. Written informed consent was obtained from the minor(s)' legal guardian/next of kin for the publication of any potentially identifiable images or data included in this article.

## Author contributions

TT and ML designed and conceptualized the report, reviewed, and revised the manuscript. XW and ND acquired and interpreted patient data and wrote the first draft of the manuscript. ZL acquired and interpreted patient data and reviewed and revised the manuscript. All authors approved the final manuscript as submitted and agreed to be accountable for all aspects of the work.

## Funding

This study was supported by Yangzhou Science Technology Project, China (YZ2019054).

## Conflict of interest

The authors declare that the research was conducted in the absence of any commercial or financial relationships that could be construed as a potential conflict of interest.

## Publisher's note

All claims expressed in this article are solely those of the authors and do not necessarily represent those of their affiliated organizations, or those of the publisher, the editors and the reviewers. Any product that may be evaluated in this article, or claim that may be made by its manufacturer, is not guaranteed or endorsed by the publisher.

## Supplementary material

Supplementary Video 1 The subject was examined at the age of 10 years. The displayed phenomenology consists of cerebellar ataxia, but no scoliosis or other musculoskeletal deformities. Signs of chorea, arm dystonia and other movements disorders have not observed. The subject walks with living support and has, over time, become more dependent on the wheelchair.
