# Supplementary figures and images for: Case report: A novel APTX p.Ser168GlufsTer19 mutation in a Chinese family with ataxia with oculomotor apraxia type 1
Source: Front Neurol. 2022 Sep 1;13:873826. doi: 10.3389/fneur.2022.873826 (PMC9479491; doi:10.3389/fneur.2022.873826)

HE:


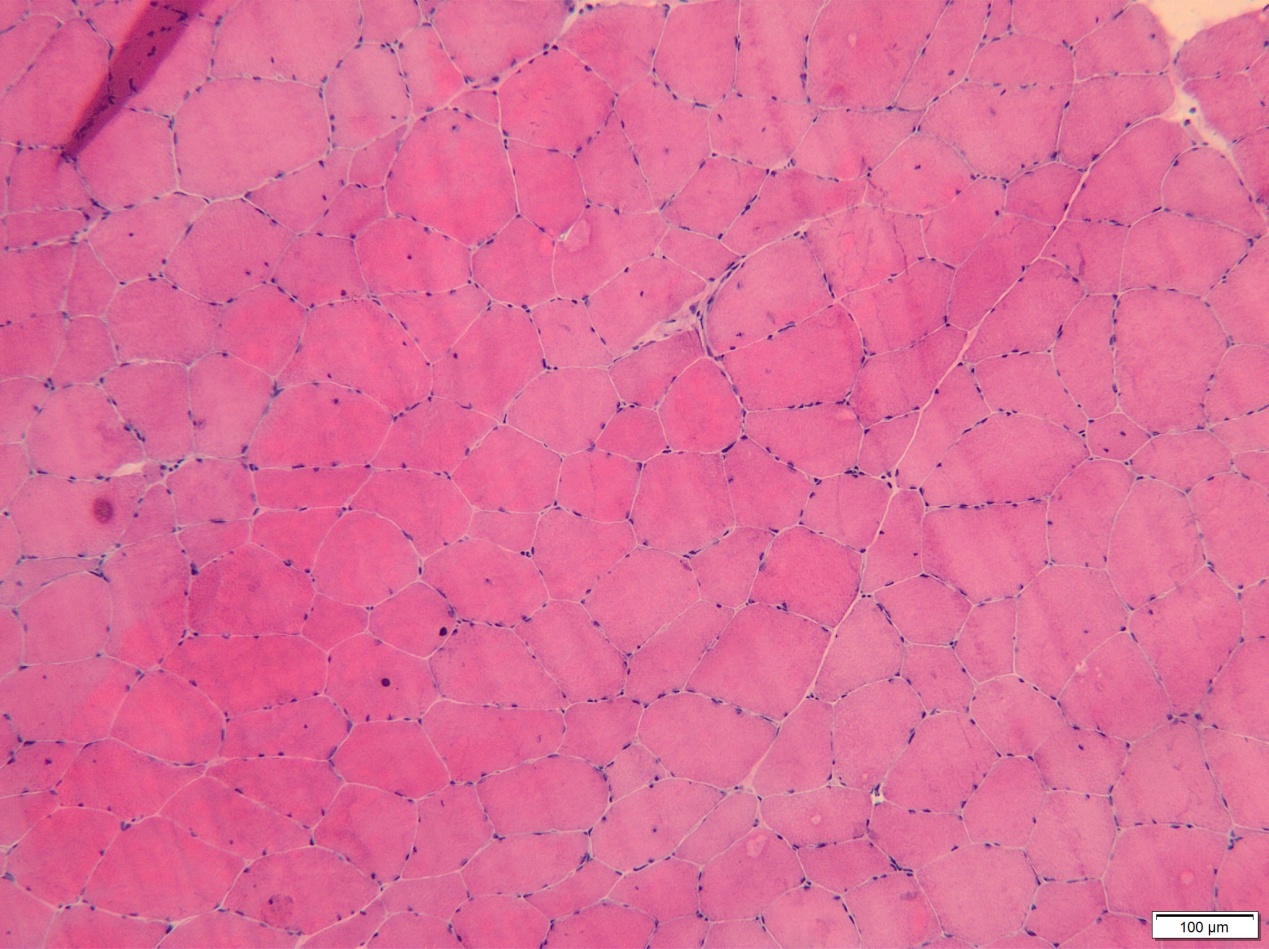


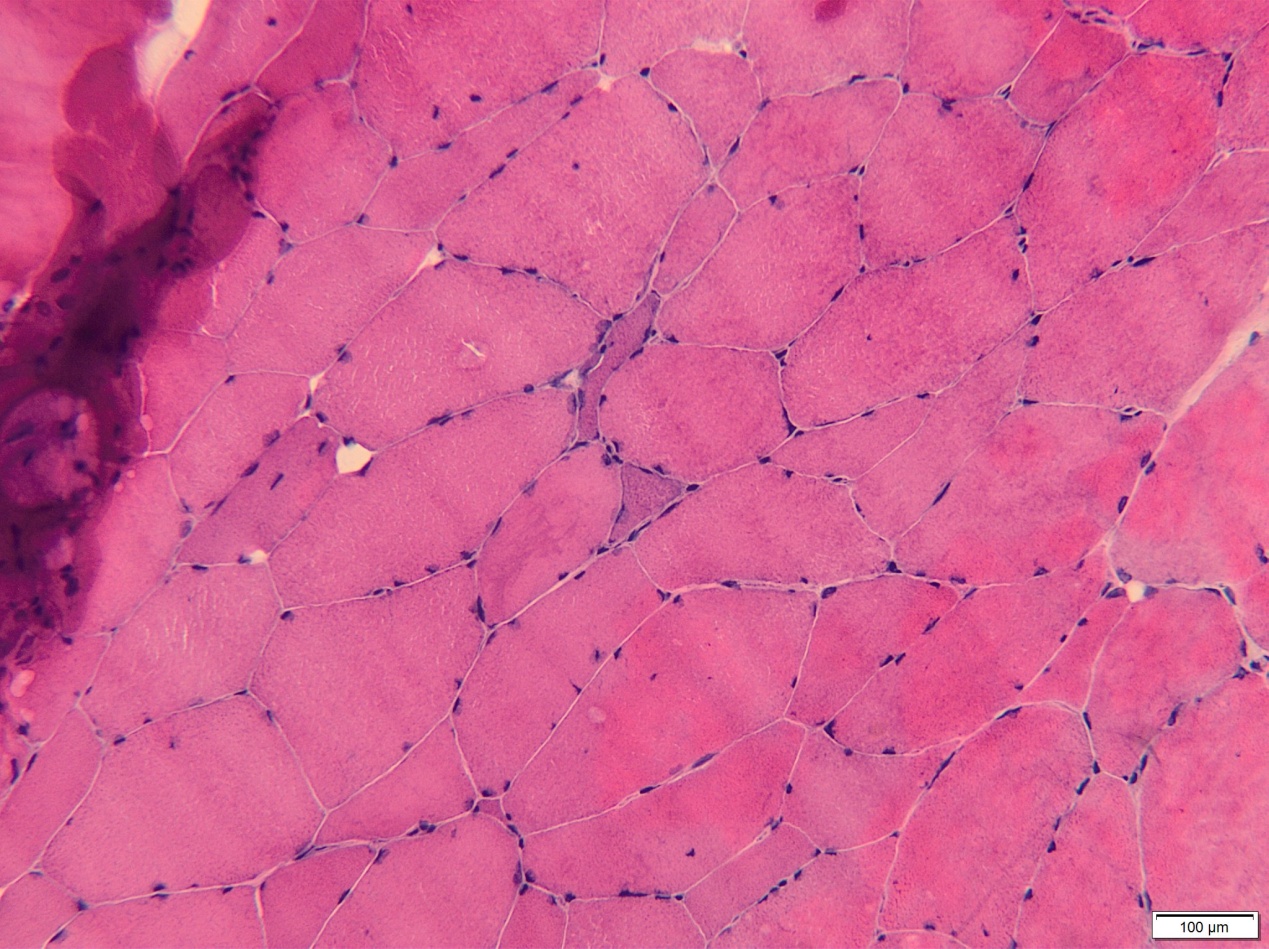


NADH


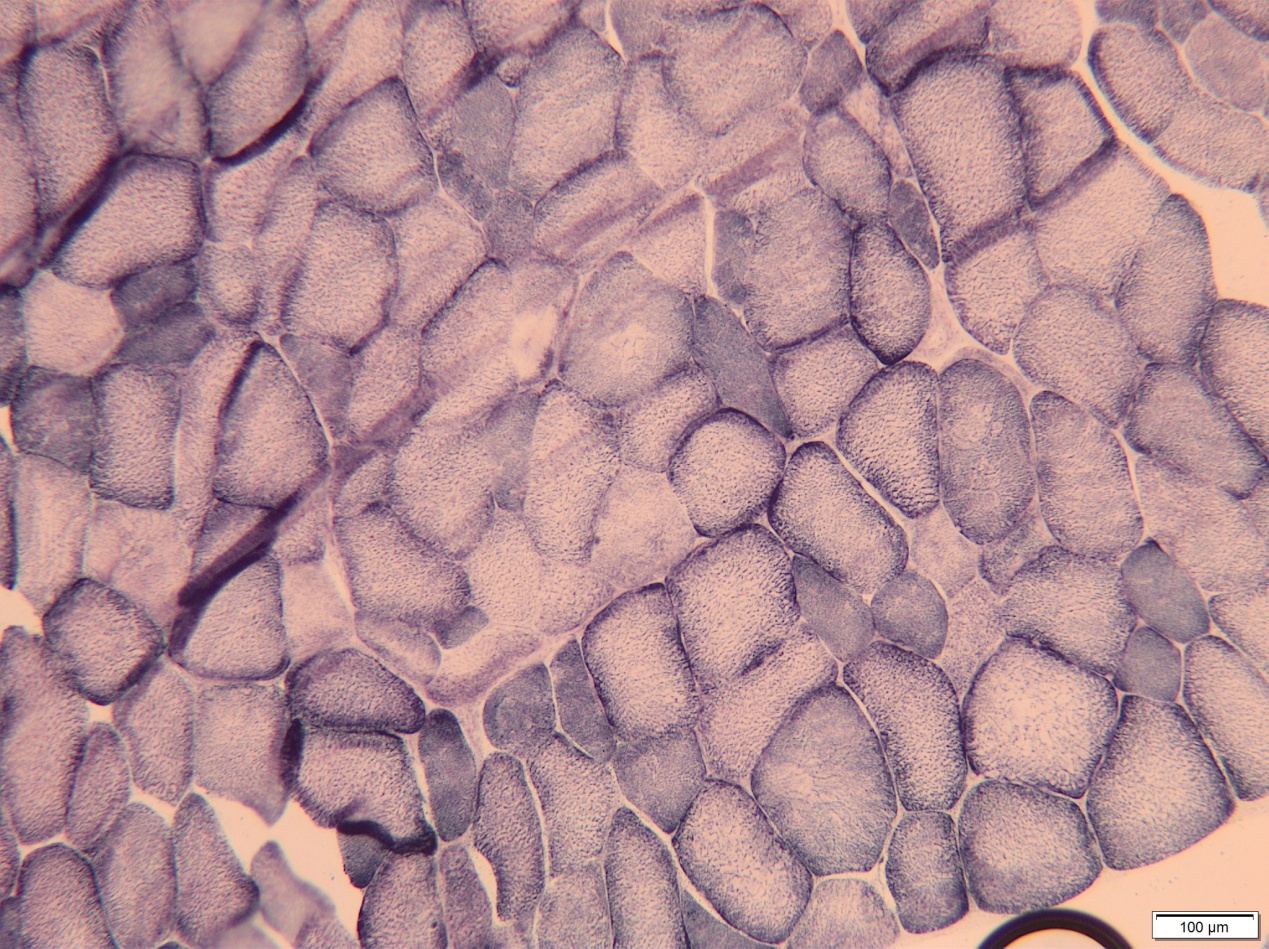


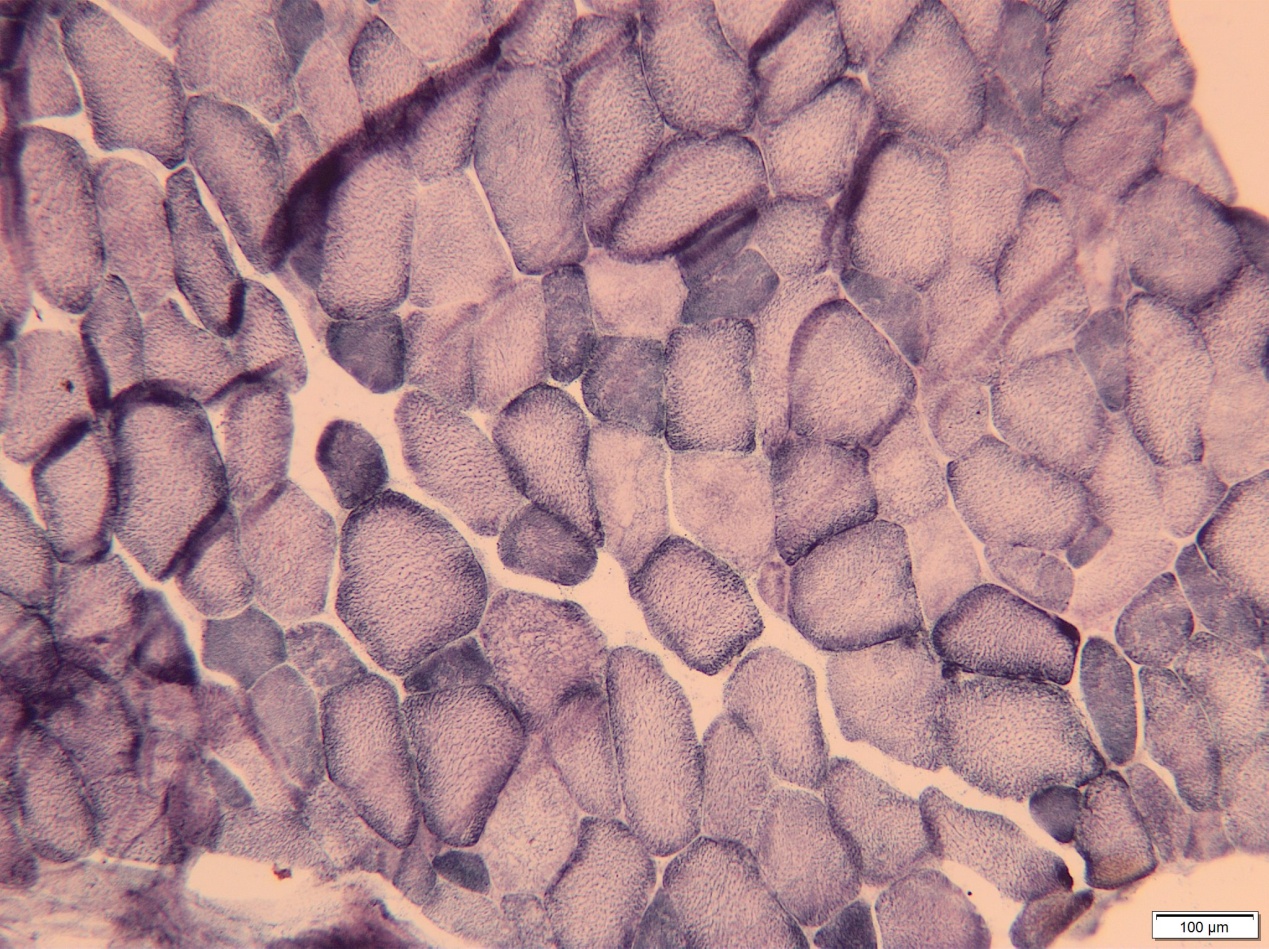


PAS:


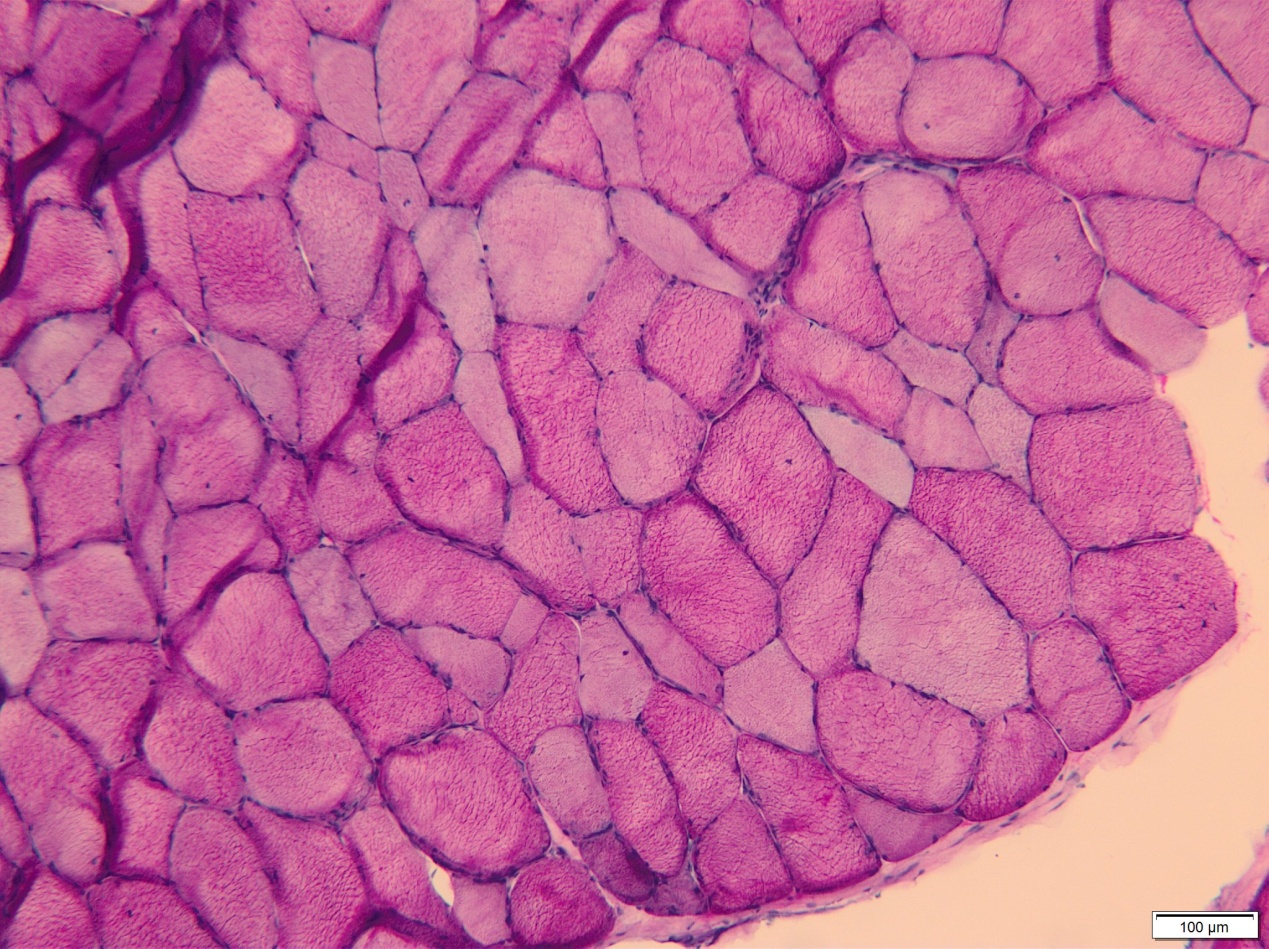


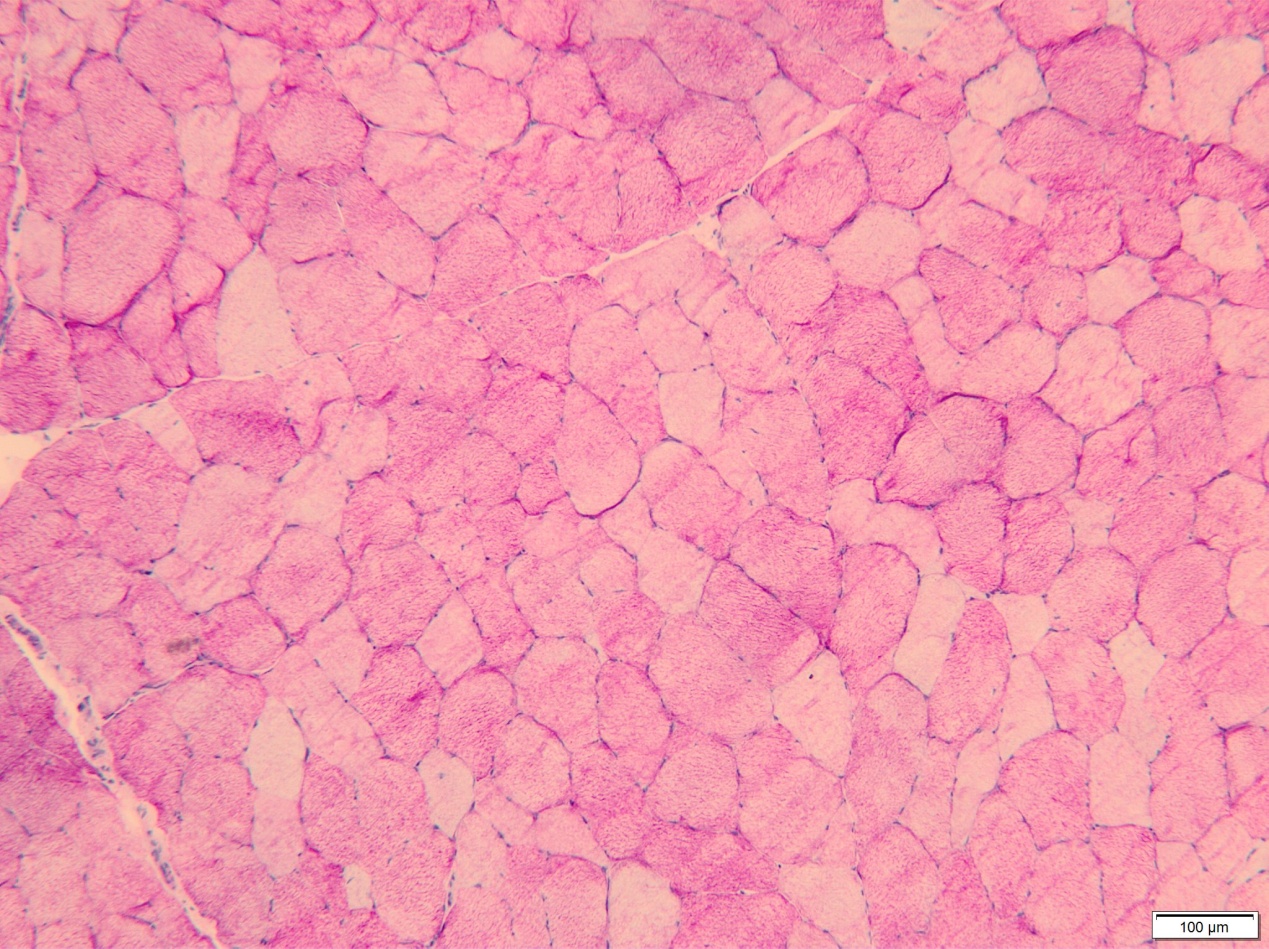


ORO:


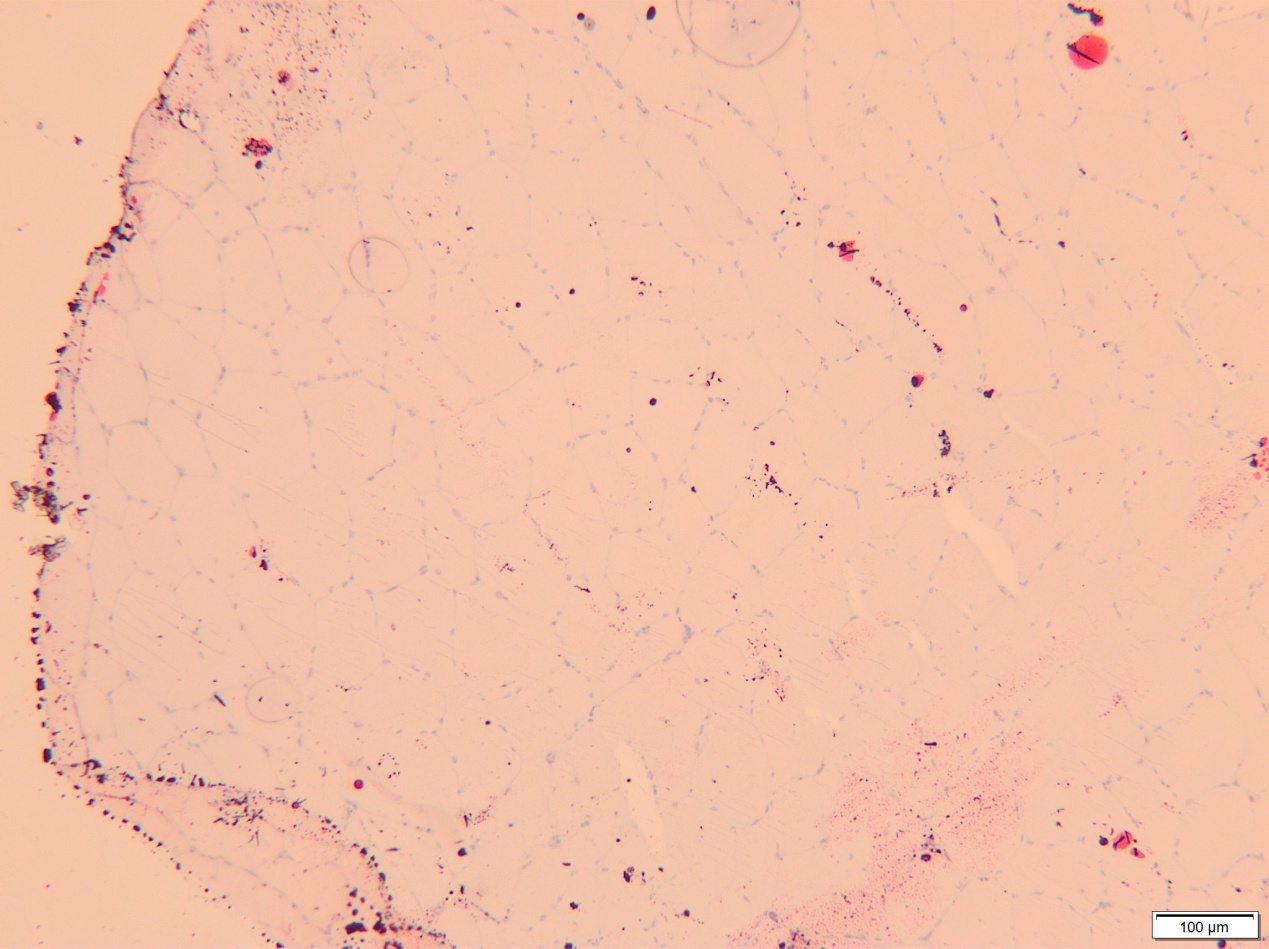


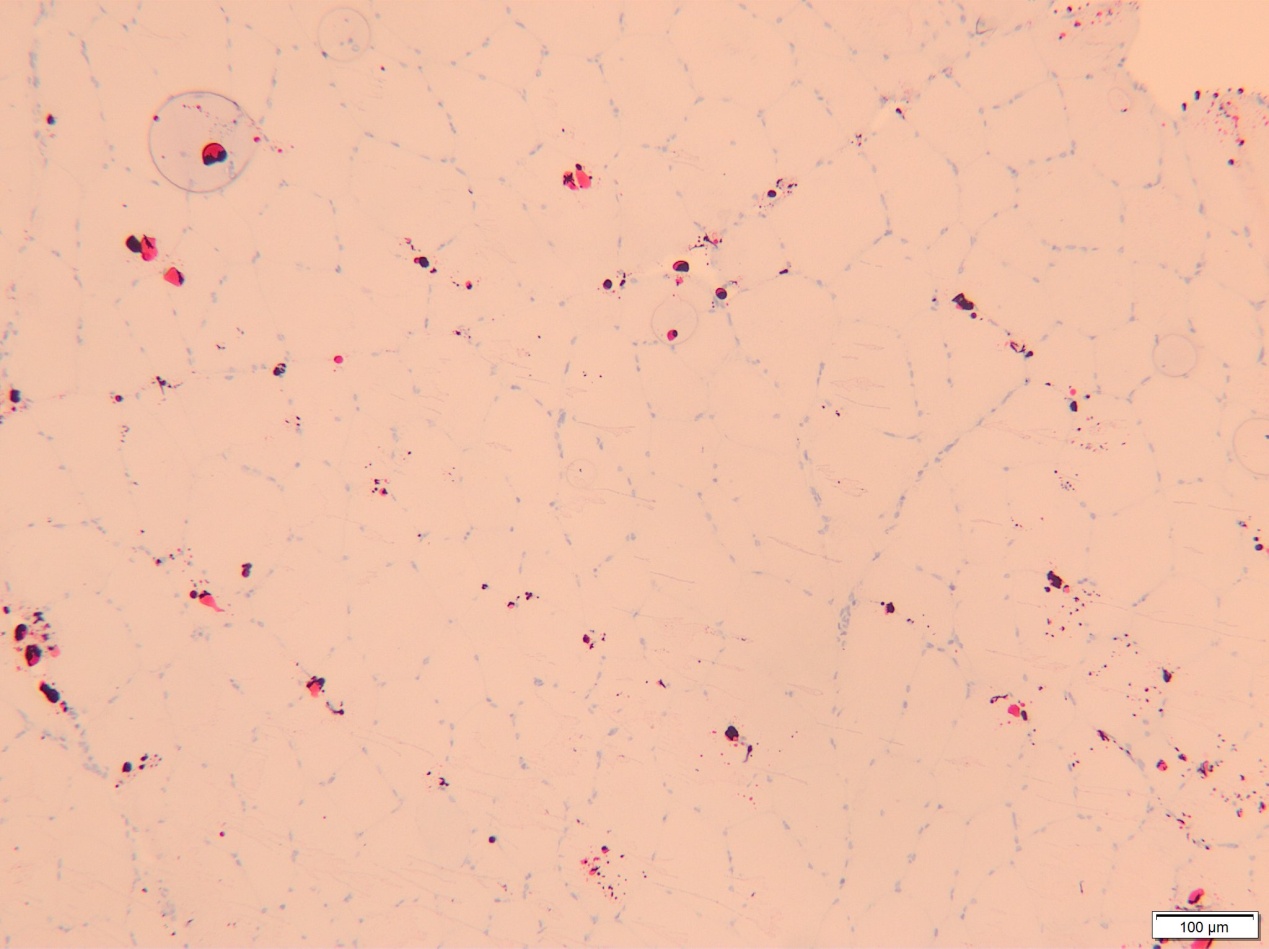


IHC

CD3:

**
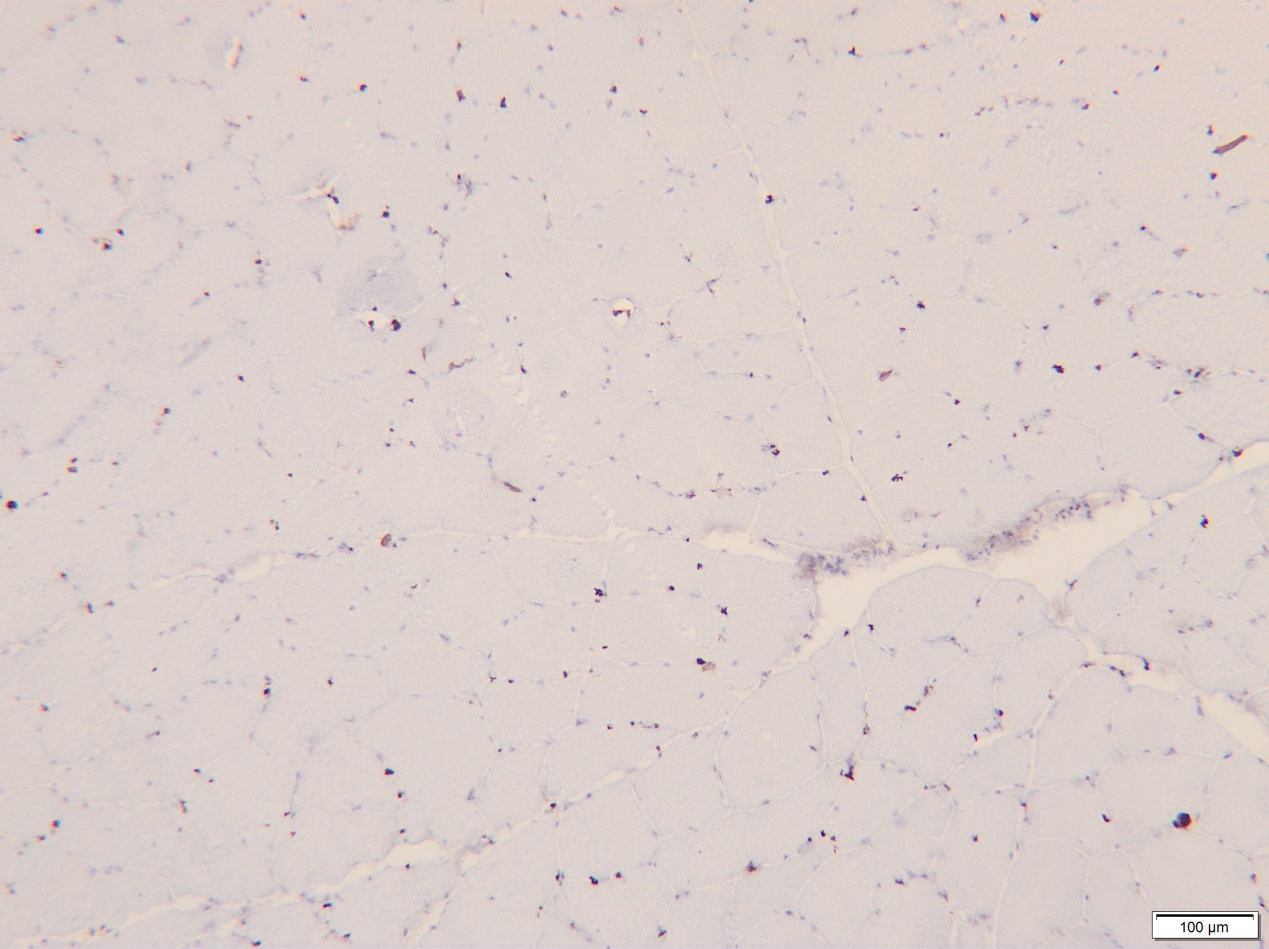
**

CD4:


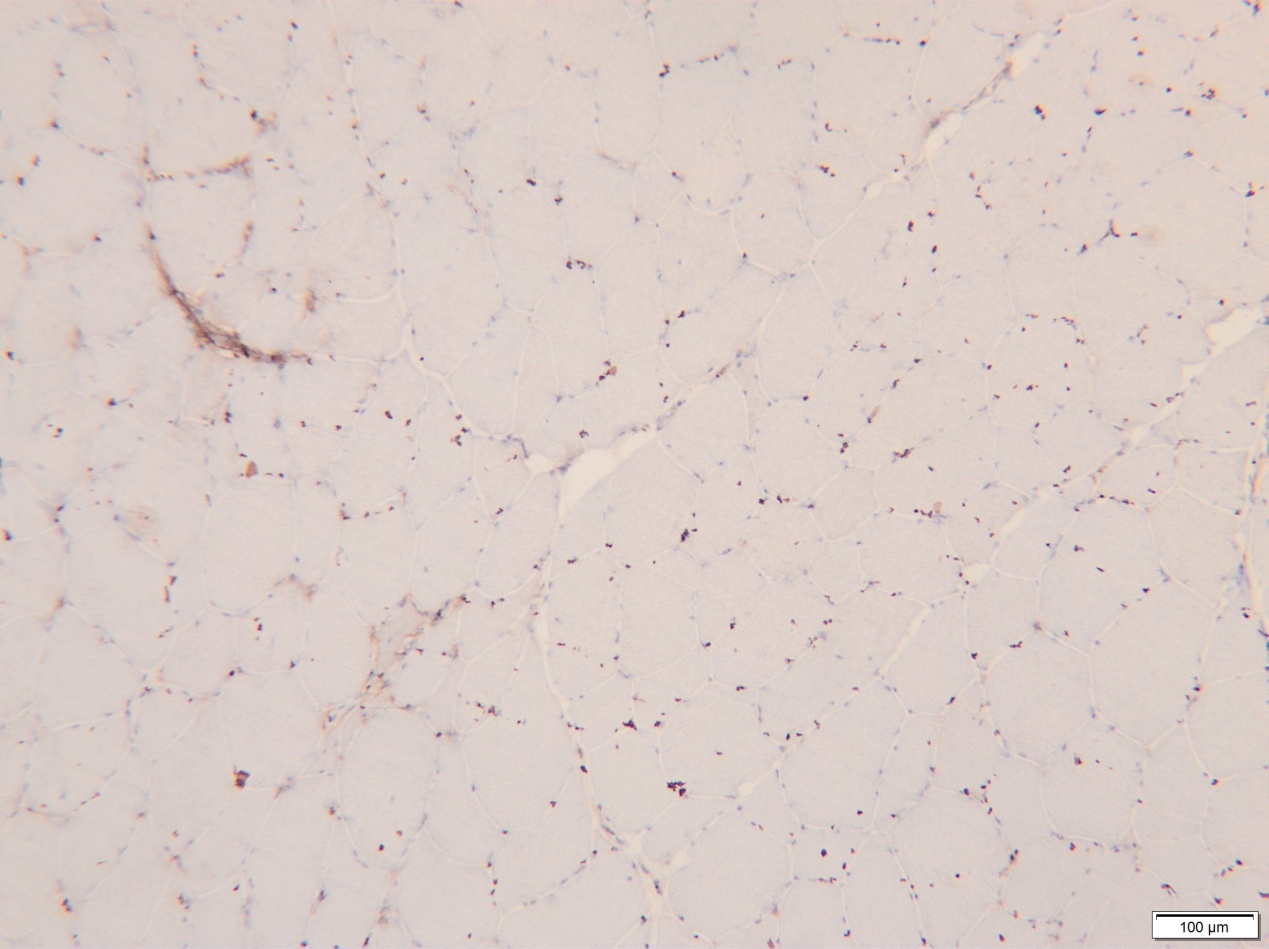


CD8:


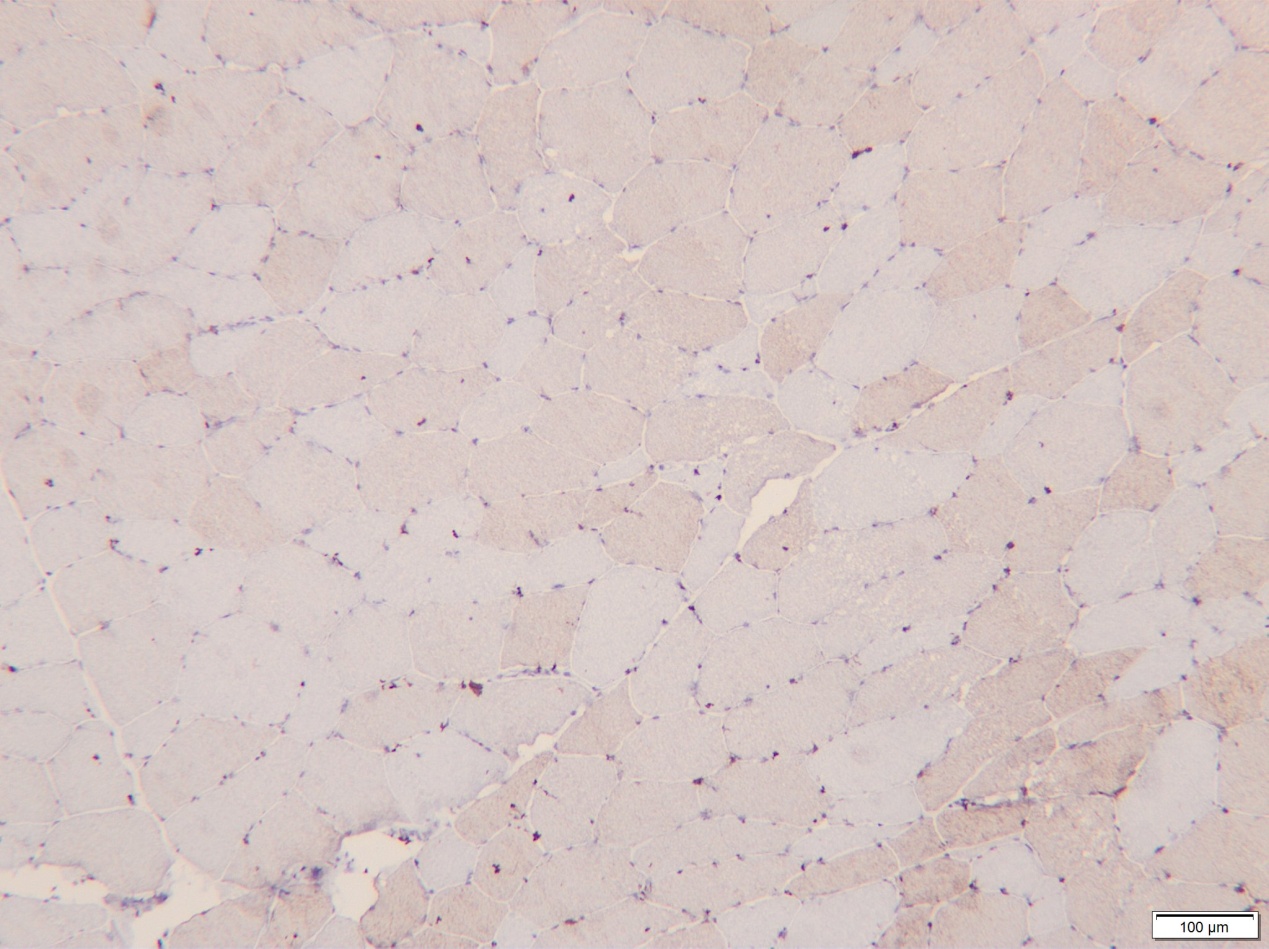


CD20:


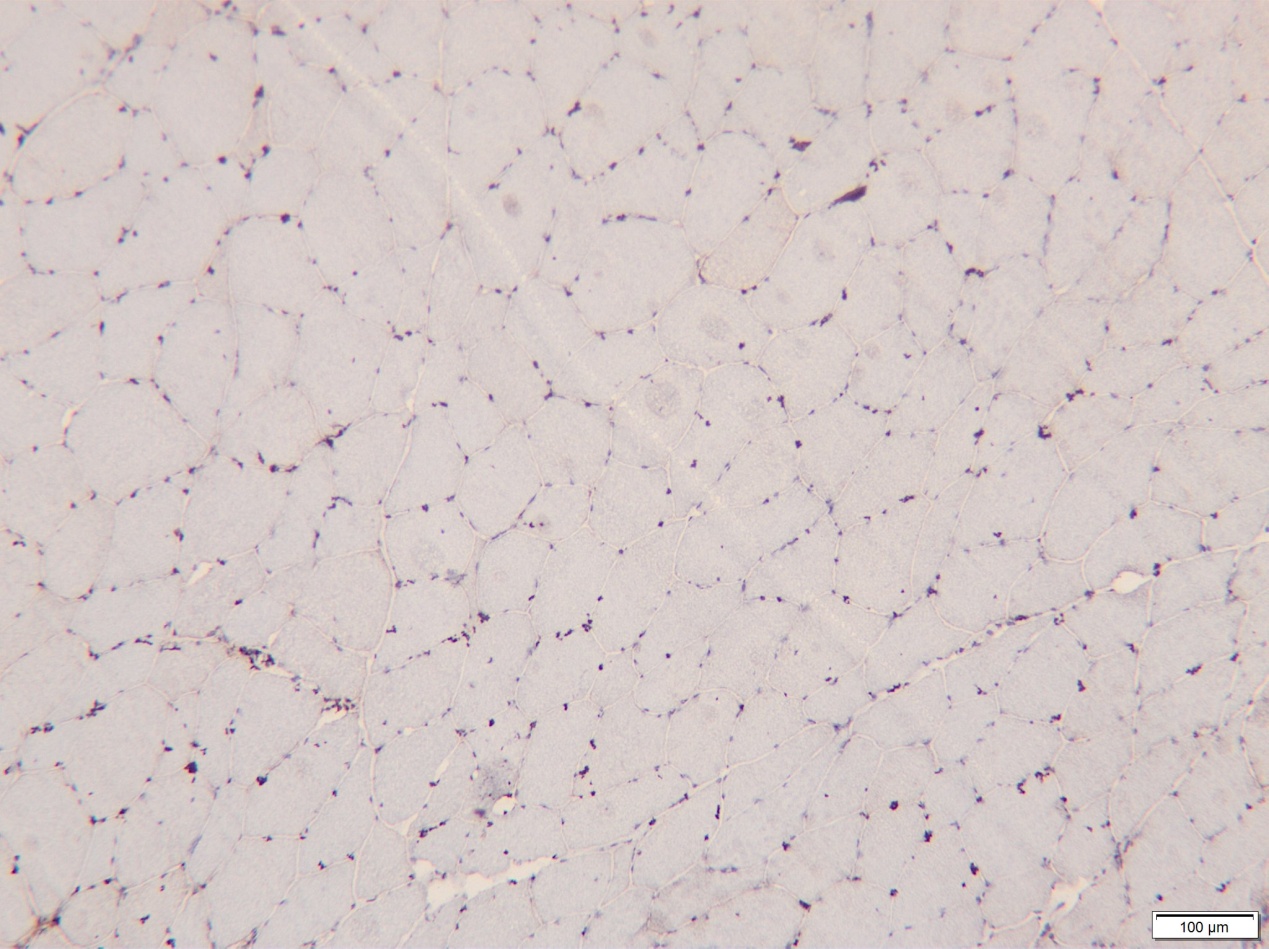


dysferlin:


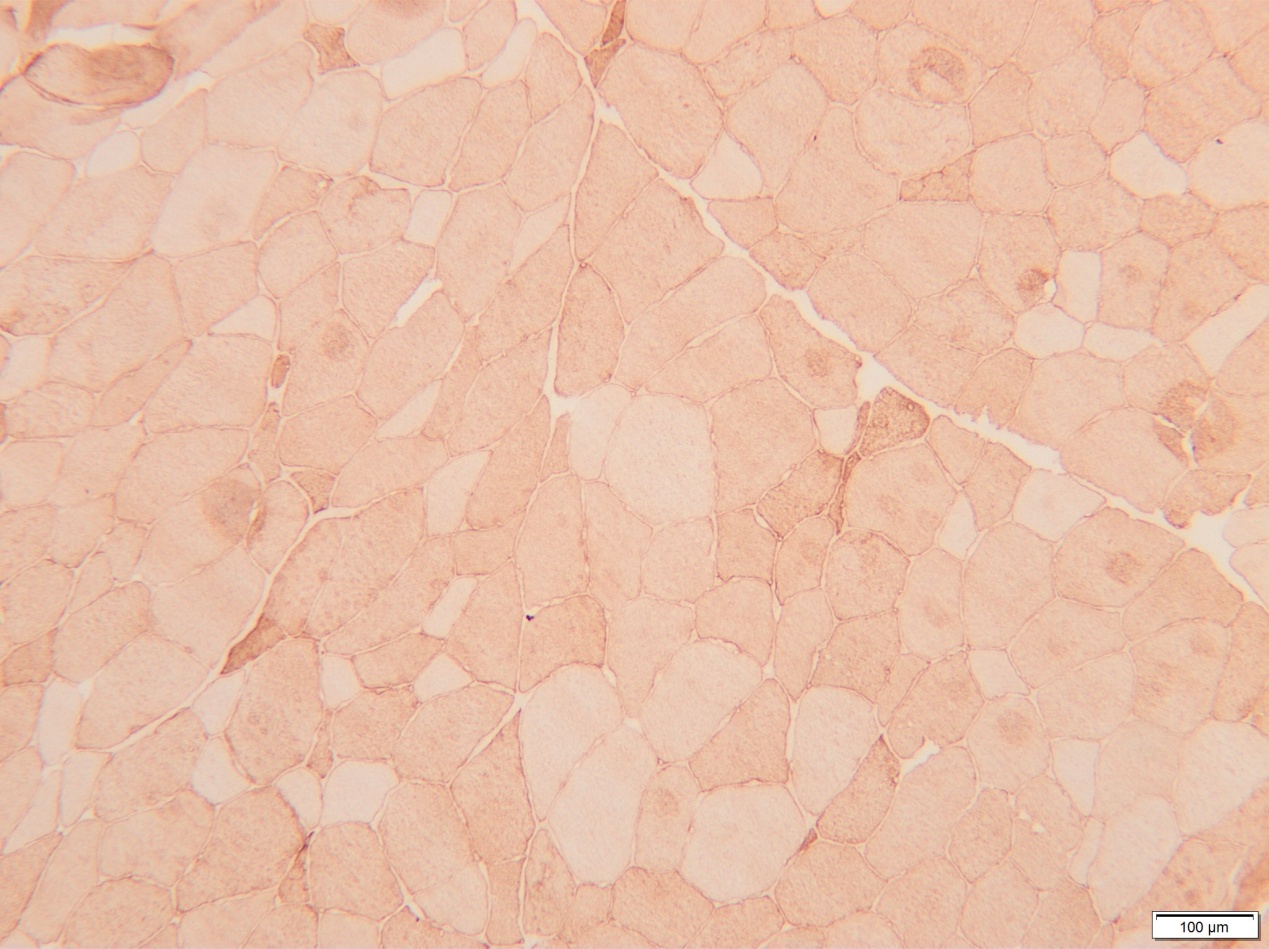


dystrophin:


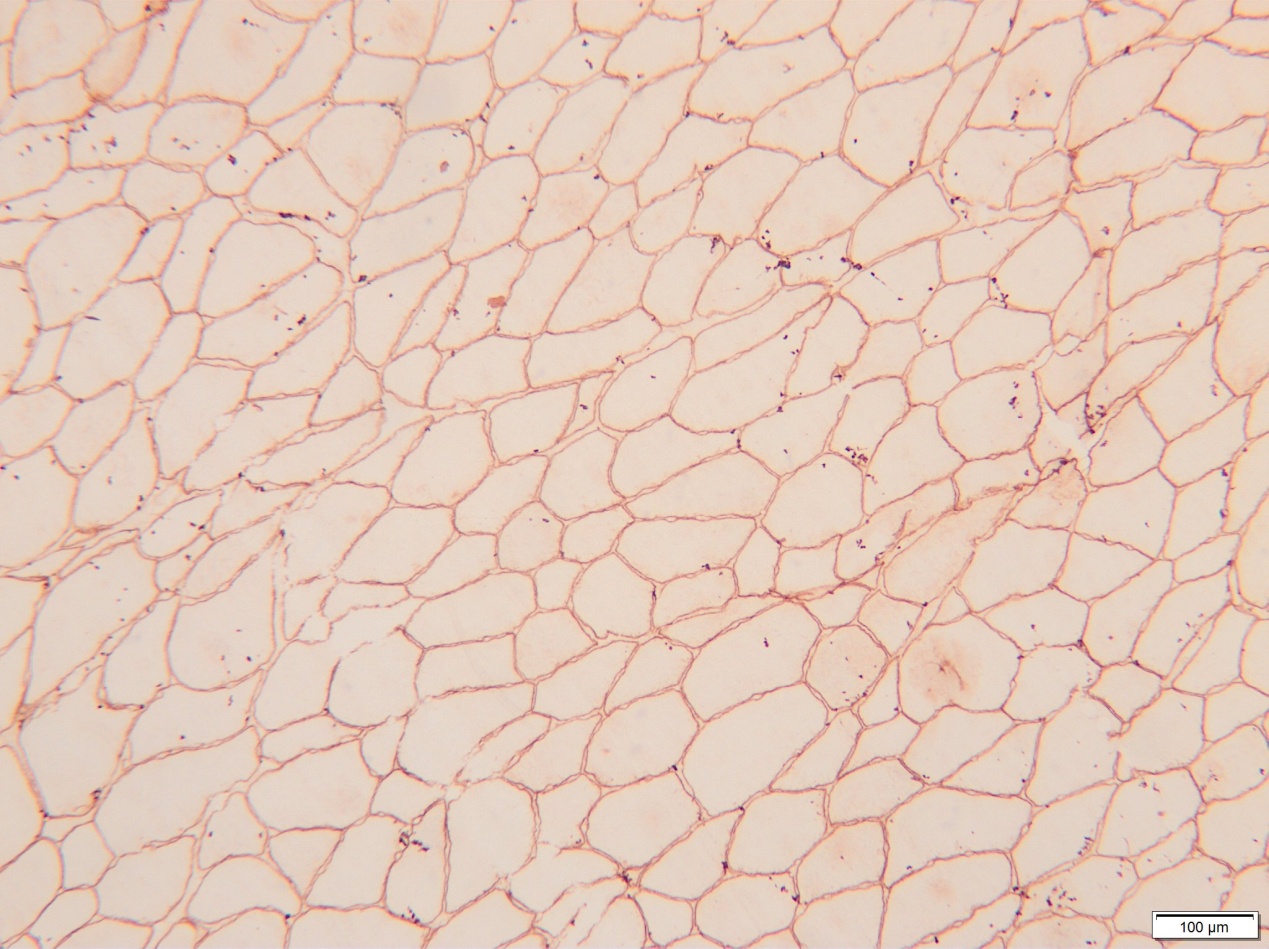


MAC:


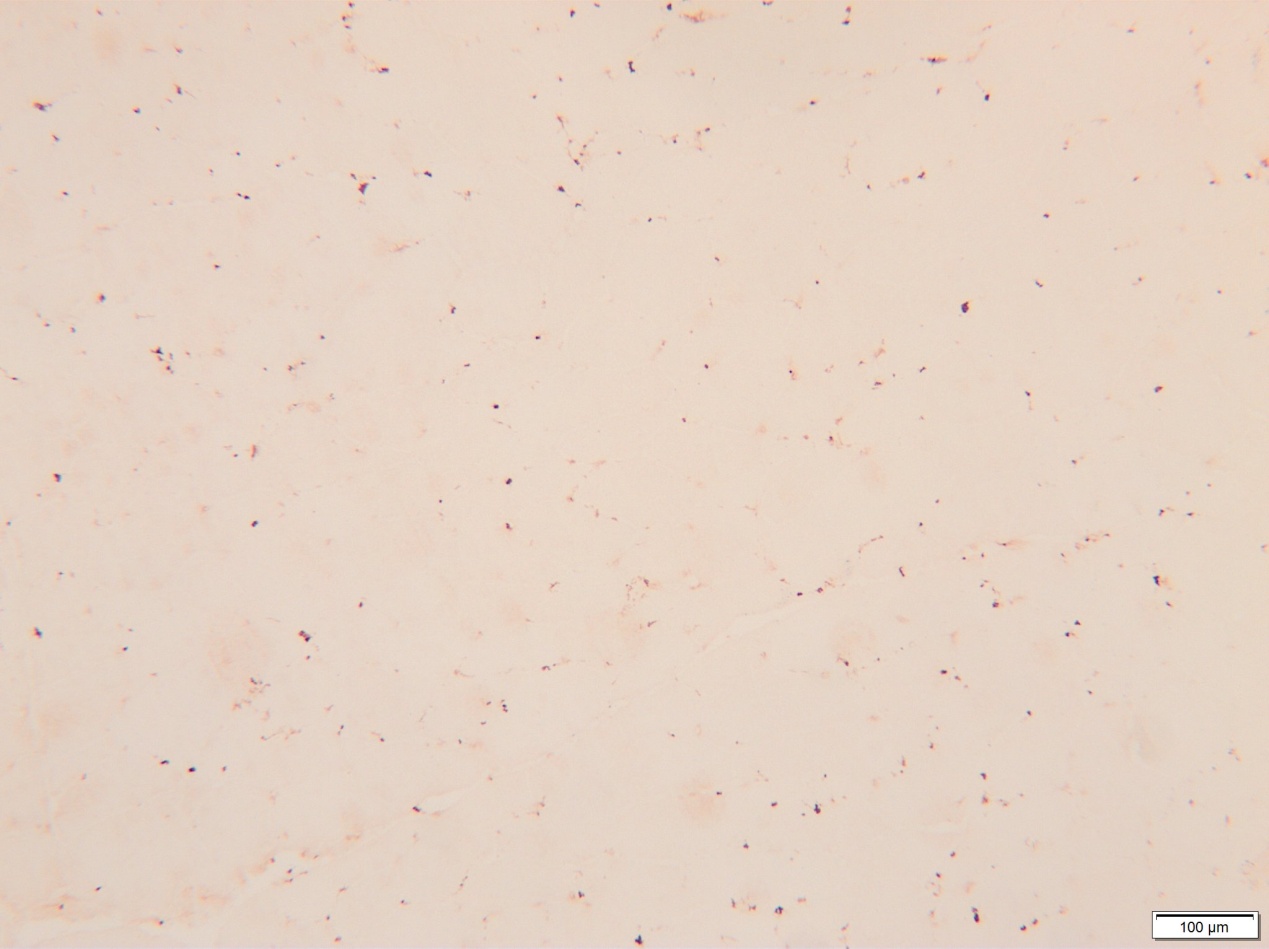


MHC-1:


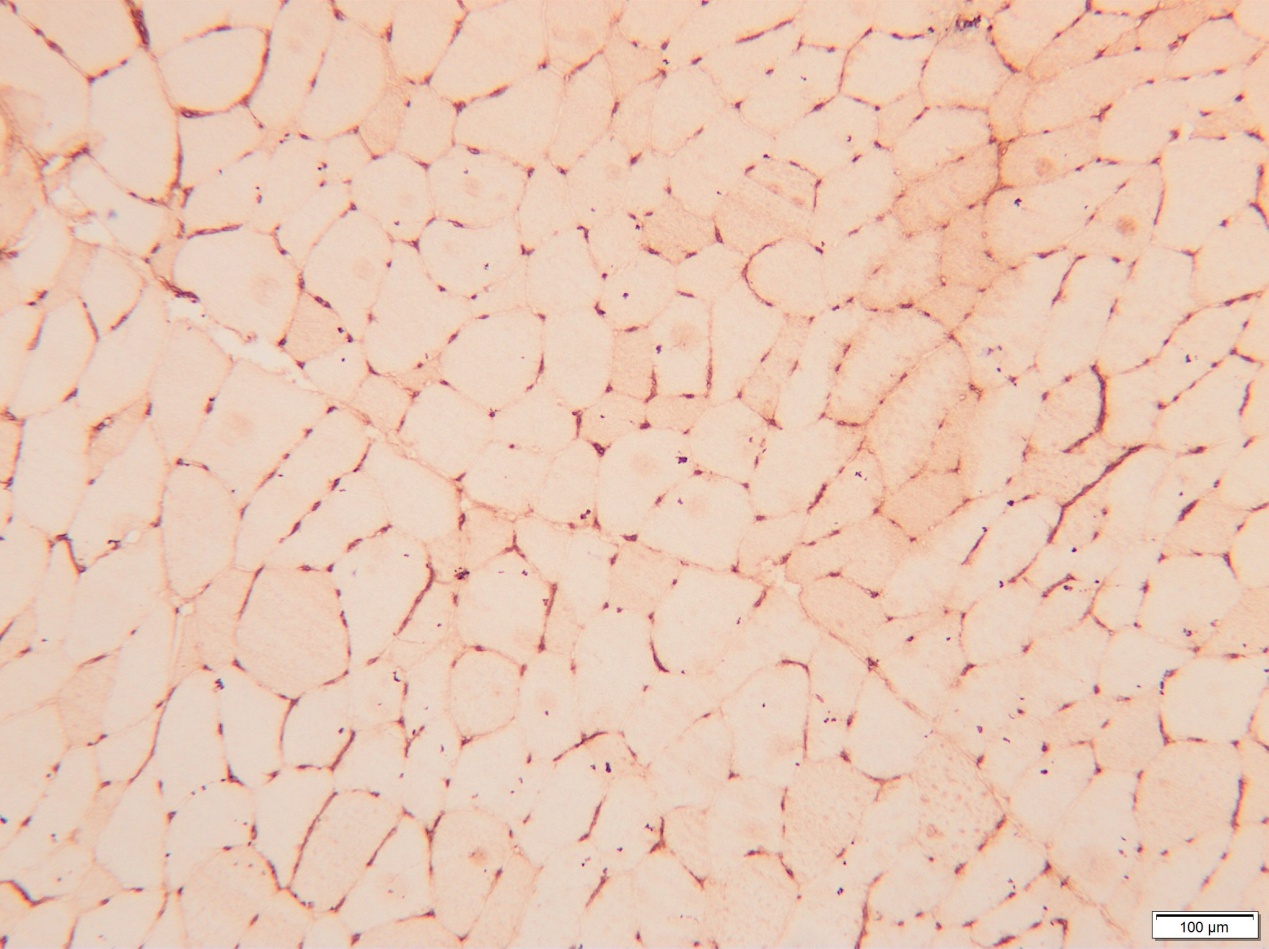


MXA:


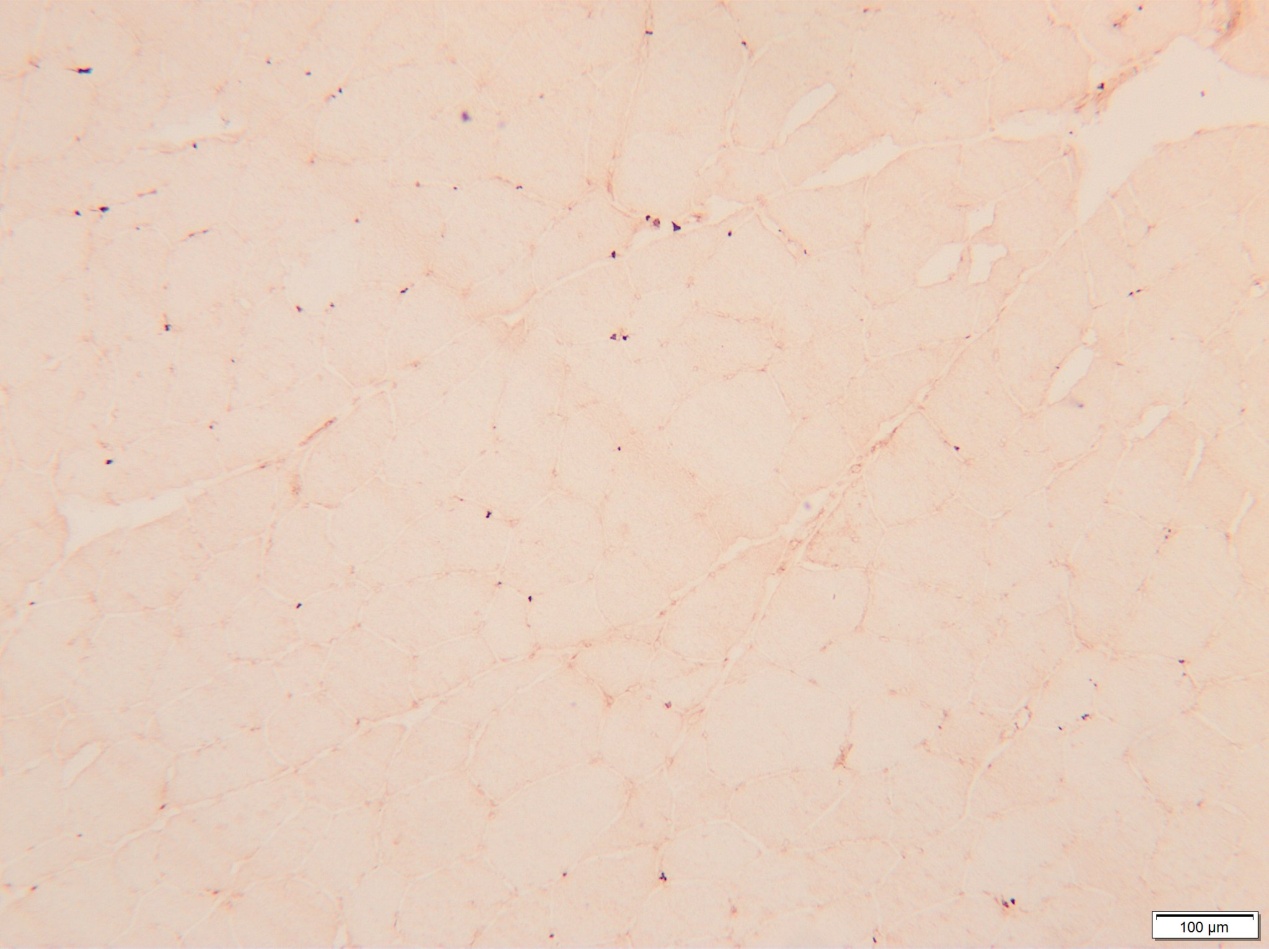

Supplement: Supplementary file 3 [file Table_2.DOCX]
